# Specialized specialists and the narrow niche fallacy: a tale of scale-feeding fishes

**DOI:** 10.1098/rsos.171581

**Published:** 2018-01-17

**Authors:** Matthew A. Kolmann, Jonathan M. Huie, Kory Evans, Adam P. Summers

**Affiliations:** 1 Friday Harbor Laboratories, University of Washington, 620 University Road, Friday Harbor, WA 98250, USA; 2 School of Aquatic and Fishery Sciences, University of Washington, 1122 NE Boat St, Seattle, WA 98195, USA; 3 College of Food, Agricultural and Natural Resource Sciences, University of Minnesota, 1987 Upper Buford Circle, St Paul, MN, USA

**Keywords:** lepidophagy, Characiformes, grazing, paedomorphosis, mucophagy, pterygophagy

## Abstract

Although rare within the context of 30 000 species of extant fishes, scale-feeding as an ecological strategy has evolved repeatedly across the teleost tree of life. Scale-feeding (lepidophagous) fishes are diverse in terms of their ecology, behaviour, and specialized morphologies for grazing on scales and mucus of sympatric species. Despite this diversity, the underlying ontogenetic changes in functional and biomechanical properties of associated feeding morphologies in lepidophagous fishes are less understood. We examined the ontogeny of feeding mechanics in two evolutionary lineages of scale-feeding fishes: *Roeboides*, a characin, and *Catoprion*, a piranha. We compare these two scale-feeding taxa with their nearest, non-lepidophagous taxa to identify traits held in common among scale-feeding fishes. We use a combination of micro-computed tomography scanning and iodine staining to measure biomechanical predictors of feeding behaviour such as tooth shape, jaw lever mechanics and jaw musculature. We recover a stark contrast between the feeding morphology of scale-feeding and non-scale-feeding taxa, with lepidophagous fishes displaying some paedomorphic characters through to adulthood. Few traits are shared between lepidophagous characins and piranhas, except for their highly-modified, stout dentition. Given such variability in development, morphology and behaviour, ecological diversity within lepidophagous fishes has been underestimated.

## 1. Introduction

Better minds than ours have pointed out the link between feeding morphology and diet, and many of the archetypes in evolutionary study are examples of the powerful links between anatomy, performance and ecology. There are some cases that imply that the greater the specialization the more finely-tuned the morphology. There are cichlids in African rift lakes that allegedly specialize in eating the eyes of other cichlids [1,2]. These fishes and sympatric scale-eating cichlids maintain a handedness polymorphism that allows half the population to deliver nasty surprises from the right side of prey while the other half operates on the left side [3]. Ecomorphological studies rely on this link, though when linking shape to diet across an ecosystem there seems to be considerable noise in the system [4,5]. Measuring morphology may be fraught with certain errors, but it may be that the very concept of a narrow dietary niche is quite often to blame. For example, molluscivory represents a near monophagous dietary specialization associated with a narrow suite of morphological characters: large jaw muscles, and robust teeth and jaws [6,7]. But diet and morphology suffer a serious mismatch when the snail-slurping snake *Sibon* is grouped ecologically with mollusc-mauling myliobatine stingrays [8,9]; the former ratchet snails from their shells with elongate, gracile lower jaws, while the latter crush prey outright with stout jaws fused at the symphysis and pavement-like dentition.

Ecological and morphological specialization are intimately tied in parasitic organisms. Parasites and parasitoids specialize on particular prey species, and their morphologies and life histories are intimately tied to the life cycle of their hosts. Vertebrate parasites are rare relative to those in other animal lineages, but a diversity of fishes specialize in feeding on the mucus and scales of other fishes. Lepidophagous fishes are represented by at least 37 genera of fishes, with this strategy evolving multiple times independently [10,11]. The success of this feeding strategy may lie in it being the carnivorous equivalent of grazing: a fish can be parasitized many times and simply grow the scales and mucus back. Scale-feeders are common in tropical marine and freshwater systems where prey species density and richness are high [3,12–15]. These vertebrate parasites have been associated with some of the fastest rates of morphological evolution recorded in vertebrates, presumably given radically specialized behaviours and morphologies [11].

As with other parasites, scale-feeders show changes in autecology with different stages in their life history and the density of their prey. The degree of lepidophagy within and between species ranges from facultative to obligatory, as well as varying with ontogeny and seasonality, in relation to prey density [16–18]. For example, crescent grunters (*Terapon jarbua*) feed increasingly more on scales in addition to whole fishes, crustaceans and ectoparasites, throughout their ontogeny [19]. Other lepidophagous fishes consume only scales, and do so throughout their entire ontogeny, such as Bahamian pupfishes (*Cyprinodon desquamator*) [11] and the bucktooth tetra (*Exodon paradoxus*) [20]. The phylogenetic diversity of these scale-feeding fishes raises the question: are all lineages converging on similar feeding morphologies and behaviours for scale-feeding? Are scale-feeders overtly similar as a guild and is lepidophagy an ecologically-singular construct?

**Figure 1.**
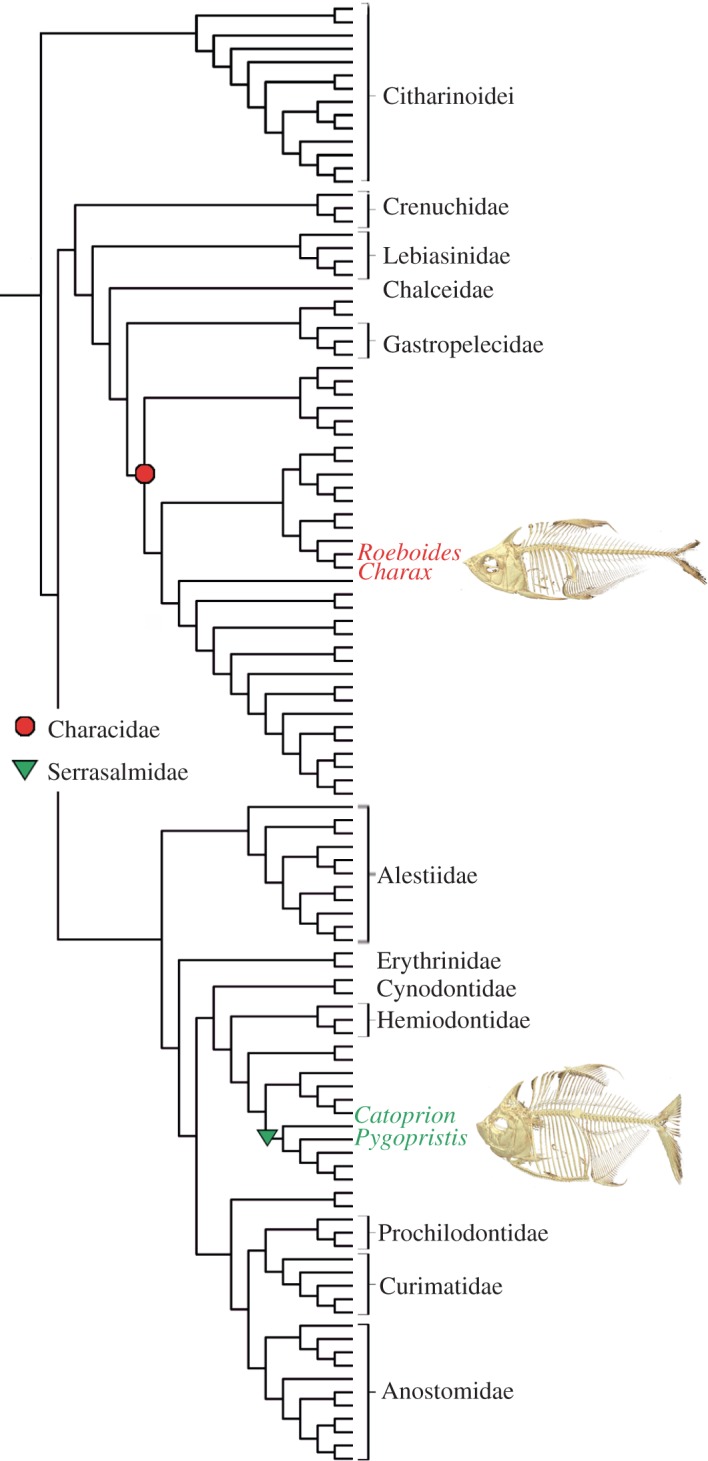
Phylogeny of characiform fishes, modified from Arcila *et al*. [21] to show the evolutionary separation of serrasalmid scale-feeders from characin scale-feeders.

**Figure 2.**
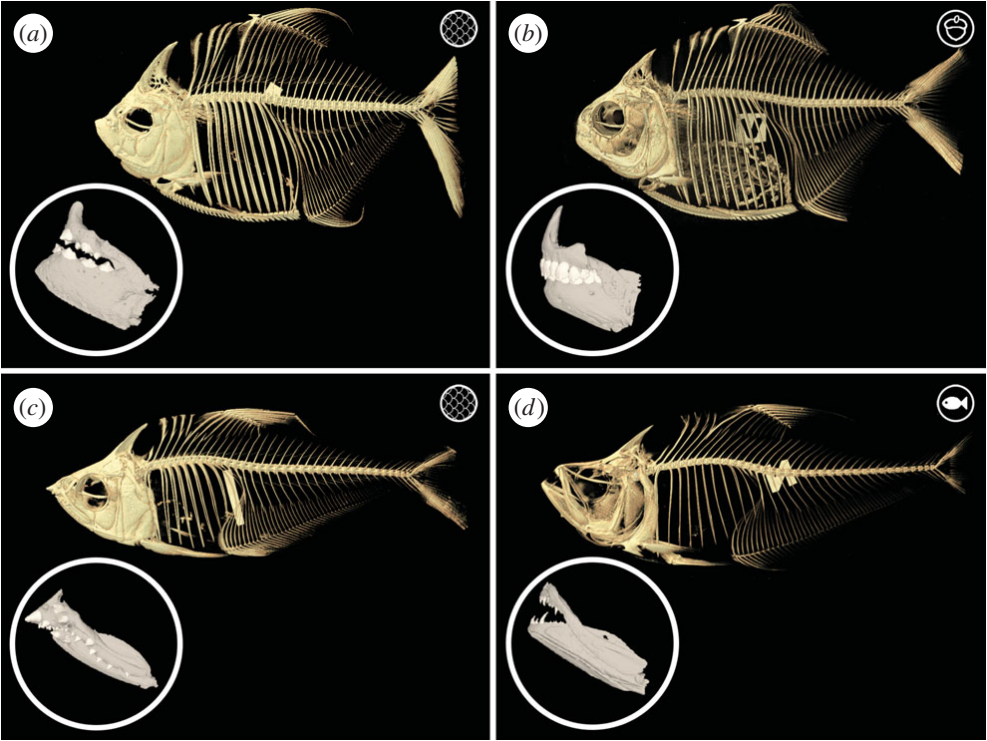
Lateral view of the reconstructed μCT scans for the four characiform species used in this study. In the lower left corner of each panel is the isolated jaw of each species. (*a*) *Catoprion mento*, (*b*) *Pygopristis denticulata*, (*c*) *Roeboides affinis*, (*d*) *Charax* cf. *pauciradiatus*.

Using both sister-species and ontogenetic comparisons, we assess developmental and functional themes in lepidophagous characiform fishes (figure 1). We used micro-computed tomography (μCT) scanning, coupled with iodine-enhanced contrast staining (diceCT) [22] to examine two independent lineages of scale feeding characiform fishes from the Neotropics. We compared the scale-eating characin (*Roeboides affinis*) and the closely related (and similarly sized) ixha (*Charax* cf. *pauciradiatus*). The ixha is a dietary generalist its whole life while the aptly named scale-eating characin does just that from transformation to adulthood [23–25]. We also compared the obligate, lifelong scale eater *Catoprion mento* (wimple piranha) and the lobe-toothed piranha (*Pygopristis denticulata*), which eats scales when young, but transitions to more complete consumption of other fishes and plants as adults [26–28] (figure 2). This system gives us two species that only eat scales and one species that only eats scales as a juvenile to compare with a non-scale-feeding species and a non-scale-feeding adult. Our objectives were: (i) to contrast the gross morphology of scale-feeding (*Catoprion* and *Roeboides*) with non-scale-feeding relatives (*Pygopristis and Charax*, respectively); (ii) to determine whether some aspects of feeding morphology augment feeding performance in lepidophagous taxa; (iii) to assess morphological convergence in the lepidophagous fishes; and (iv) to assess whether the lepidophagous ‘niche’ in these two lineages is a useful construct.

## 2. Methods

### 2.1. Specimen acquisition and micro-CT scanning

We used μCT scanning to visualize and measure the cranial morphology of ontogenetic series of four species. *Catoprion mento* (*n* = 11) specimens were loaned from the Royal Ontario Museum (Toronto, CA) and *Roeboides affinis* (*n* = 8), *Pygopristis denticulata* (*n* = 8), and *Charax* cf. *pauciradiatus* (*n* = 8) specimens were obtained from the Auburn University Museum (Auburn, USA). For iodine-contrast staining of each ontogenetic series, we used a subset (*n* = 6–8) of the previously scanned specimens. Prior to scanning, specimens were tagged with a radiopaque label and then fishes of similar sizes were wrapped in 70% ethanol-soaked cheesecloths and packed tightly into a PLA (polylactic acid)-plastic cylinder.

Specimens were scanned using the Bruker Skyscan 1173 at the Karel F. Liem Bio-Imaging Center at Friday Harbor Laboratories at 65 kV and 123 µA with a voxel size ranging from 17.1 to 33.5 µm. The nature of CT scanning only allows X-ray imaging to capture dense material such as bone, dentine and enamel; however, soft tissues such as muscle and tendon can be visualized through contrast-staining with a chemical agent such as iodine. We modified the iodine contrast-staining method of Gignac & Kley [22], to visualize muscle tissue. All specimens were soaked in an aqueous solution of 3% Lugol's iodine (i.e. 0.75% I_2_ and 1.5% of KI) for 12 h or until specimens were completely stained. Specimens were then patted dry and prepared for CT scanning as described above.

### 2.2. Functional morphology of the feeding apparatus

Reconstructed scans were converted to .dcm format and exported into the CT segmentation program, Horos (The Horos Project, 2015 http://www.horosproject.org/). We used the 3D-MPR mode in Horos to measure functional aspects of the cranial morphology of these fishes, including: (i) tooth aspect-ratio for comparing tooth shape and robustness; (ii) occlusional offset, an indicator of slicing or crushing jaw action; (iii) anterior (AMA) and posterior jaw mechanical advantage (PMA), measurements of jaw leverage or force transmittance to prey (electronic supplementary material, figure S1); (iv) jaw adductor muscle cross-sectional area (CSA), a proxy for muscle force generation; and (v) second moment of area of the jaws, a proxy for jaw stiffness in either vertical or lateral bending.

Tooth aspect ratio was calculated as the maximum tooth height divided by its perpendicular maximum tooth width. We measured three undamaged, mature teeth that were representative of overall tooth shape. To measure occlusional offset, we first drew a line from the tip of the anterior most tooth to the posterior most tooth in the lower jaw [29]. Then we measured the orthogonal distance from that axis to the jaw joint. We measured mechanical advantage (a ratio which evaluates trade-offs between jaw leverage and jaw-closing speed), in piranhas from the jaw joint to the insertion point of the adductor mandibulae tendon [30]. However, for the characids we approximated the centre of the concave area in the mandible and used that as the insertion point as muscle-scarring was not immediately obvious. Anterior and posterior out-lever were measured from the jaw joint to the middle of the anterior and posterior tooth, respectively. After iodine-staining, we could clearly observe and segment the primary jaw adductor muscles in Horos. We looked at the primary jaw closing muscle, the adductor mandibulae (only the alpha division), and measured the cross-sectional area of those muscles [31]. For measurements of muscle CSA, we first determined myofibre directionality, made a digital slice through the muscle perpendicular to fibre direction at the estimated centre of muscle mass, and finally measured this area with the polygon tool in Horos. Jaw height and width were measured at two different regions (0%, just adjacent to symphysis, and 90%, at jaw joint) along the central axis of the lower jaw. For calculations of second moment of area, jaw height was considered the major axis while jaw width was considered the minor axis. These values were used in the equation:

$$I=\frac{\pi }{4}ab^{3},$$

where *a* is jaw height (major axis length) and *b* is jaw width (minor axis length). We used size-corrected jaw height as a proxy for jaw stiffness in the PCA.

### 2.3. Statistical analysis

We used analysis of variance (ANOVA) to contrast gross trends in skull skeletal architecture and muscle CSA among lepidophagous and non-lepidophagous taxa over their ontogeny. We size-corrected these data by regressing each morphological trait against each fish's standard length (SL) for ANOVA, calculated the residuals, and used these values as our size-corrected morphometric measures. Since ratios and angular measurements are proportions, and therefore naturally size-corrected, we did not transform mechanical advantage or aspect ratios [32]. We also examined the scaling relationships of how the above, measured morphometric traits changed over ontogeny using reduced major axis regression (RMA) [33] using the *lmodel2* package [34]. We used the *smatr 3* package [35] to confirm significant differences between these ontogenetic slopes. Scaling data were log-transformed prior to scaling analyses, excluding ratios as above [32]. We also visualized the functional feeding morphospace for all species using a principal components analysis. All statistical tests were analysed using R (www.r-project.org)

## 3. Results

### 3.1. Differences in feeding morphology between sister taxa

The two piranhas differed significantly for most traits, including tooth aspect ratio, anterior mechanical advantage, posterior mechanical advantage, jaw length, occlusional offset and adductor muscle CSA. ANOVAs of tooth aspect ratio showed that *P. denticulata* had longer and narrower teeth than *Catoprion*, which had stouter more spatulate teeth (0.96 ± 0.04 s.e. versus 0.85 ± 0.02 s.e. respectively, *p* = 0.013), at all sizes. *Catoprion* deviated from more scissor-like jaw action, exhibiting greater occlusional offset (3.72 mm ± 0.47 s.e.) relative to *Pygopristis* (2.59 mm ± 0.46 s.e., *p* = 0.004). *Catoprion* (10.0 mm ± 1.27 s.e.) have longer jaws than *Pygopristis* (6.04 mm ± 0.89 s.e., *p* < 0.001), which is reflected in *Catoprion* having lower jaw leverage at the anterior-most tooth compared to *Pygopristis*. The average AMA of *Pygopristis* (0.62 ± 0.01 s.e.) was similar to that of *Catoprion* (0.60 ± 0.01 s.e., 0.03, *p* = 0.035); however, in *Pygopristis* jaw leverage at the posterior of the jaws (PMA) (1.19 ± 0.02 s.e.) was less than that of *Catoprion* (1.45 ± 0.02 s.e., *p* < 0.001). Second moment of area for the jaws at the symphysis did not differ (1.04 ± 0.03 s.e. versus 0.29 ± 0.02 s.e., *p* = 0.43), but differed at the jaw joint (0.06 ± 0.03 s.e., versus 0.17 ± 0.09 s.e., *p* = 0.001) in *Catoprion* and *Pygopristis*. Finally, *Pygopristis* had significantly larger jaw adducting muscles than *Catoprion* (12.6 mm^2^ ± 3.2 s.e., 6.2 mm^2^ ± 1.9 s.e. respectively, *p* = 0.001).

Compared to the stark morphological contrast between *Catoprion* and *Pygopristis*, *Roeboides* and *Charax* did not display overt distinctions in feeding morphology. Only tooth aspect ratio, PMA and jaw length differed between these sister characid species. *Charax* generally had narrower, pointed teeth (2.22 ± 0.11 s.e.) than *Roeboides* (0.868 ± 0.01 s.e., *p* < 0.0001), which had broader, more robust teeth. Jaw length was greater in *Charax* over *Roeboides* (*Charax*: 10.32 ± 1.16 s.e., *Roeboides*: 8.64 mm ± 0.91 s.e., *p* < 0.001), evident in the noticeable overbite in the latter characid species. Occlusional offset (0.09) and anterior mechanical advantage (*p* = 0.089) were statistically indistinguishable between *Roeboides* and *Charax.* However, *Charax* had greater posterior mechanical advantage (8.66 ± 0.4 s.e.) relative to *Roeboides* (0.683 ± 0.02 s.e., *p* = 0.003). Second moment of area for the jaws differed at both the symphysis (0.03 ± 0.01 s.e. versus 0.01 ± 0.01 s.e., *p* = 0.023) and the jaw joint (0.06 ± 0.03 s.e., versus 0.18 ± 0.06 s.e., *p* < 0.001) in *Roeboides* and *Charax*. *Charax* also had significantly larger adductor muscles than *Roeboides* (2.29 mm^2^ ± 0.52 s.e., 3.07 ± 0.84 s.e., respectively, *p* = 0.006).

### 3.2. Intraspecific ontogenetic change in feeding morphology

**Figure 3.**
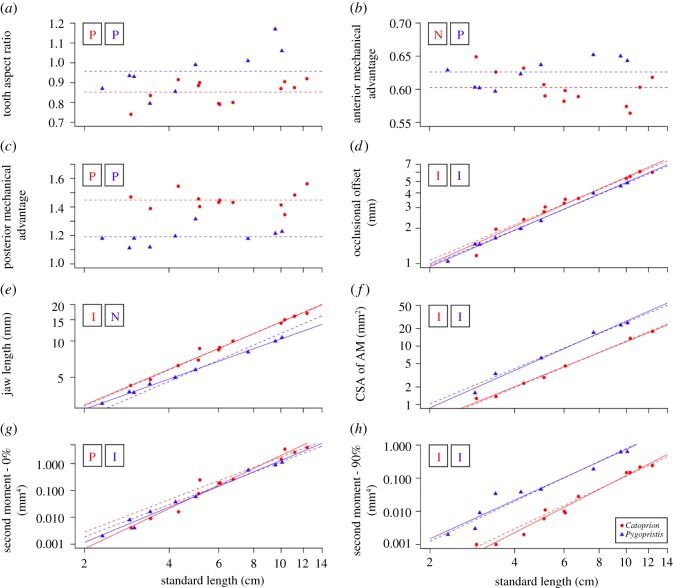
Reduced-major axis regressions of feeding morphology traits and standard length in *Catoprion mento*, and *Pygopristis denticulata* over ontogeny. (*a*) Tooth aspect ratio, (*b*) anterior mechanical advantage, (*c*) posterior mechanical advantage, (*d*) occlusional offset, (*e*) jaw length, (*f*) jaw adductor cross-sectional area, (*g*) second moment of area at the jaw joint (0%), (*h*) second moment of area at the jaw symphysis (90%). The dotted lines indicate the predicted isometric curve. Boxes represent the scaling pattern displayed by each species: N = negative allometry, P = positive allometry, I = isometric growth. Scale-feeders are outlined in red, their non-lepidophagous relatives outlined in blue.

Tooth aspect ratio scaled with positive allometry in both piranha species over ontogeny (*Catoprion*: slope = 0.15, *Pygopristis*: slope = 0.21; table 1, figure 3). However, the manner in which the teeth occlude did not deviate across ontogeny; occlusional offset showed isometric growth for both piranha species (*Catoprion*: slope = 1.05, *Pygopristis*: slope = 1.02; table 1, figure 3). In *Catoprion*, jaw length scaled isometrically, while jaw length in *Pygopristis* scaled with negative allometry, shortening relative to body length over ontogeny (*Catoprion*: slope = 0.98, *Pygopristis*: slope = 0.83; table 1, figure 3). While anterior mechanical advantage scaled with negative allometry in *C. mento*, in *P. denticulata* jaw leverage scaled positively (*Catoprion*: slope = −0.08, *Pygopristis*: slope = 0.063; table 1, figure 3). However, the slopes of these anterior mechanical advantage lines were not significantly different (*p* = 0.79; table 1). Conversely, mechanical advantage at the rear of the jaws scaled with positive allometry in both *C. mento* and *P. denticulata* (*Catoprion*: slope = 0.09, *Pygopristis*: slope = 0.09, figure 3). Scaling of second moment of area at the symphysis differed between piranhas, scaling with positive allometry in *Catoprion* but not in *Pygopristis* (*Catoprion*: slope = 4.96, *Pygopristis*: slope = 4.36, figure 3), but did not differ at the jaw joint (isometry; *Catoprion*: slope = 4.33, *Pygopristis*: slope = 3.86). Finally, the cross-sectional area of the jaw muscles grew isometrically in both *C. mento* and *P. denticulata* (*Catoprion*: slope = 1.95, *Pygopristis*: slope = 2.13; table 1, figure 3).

**Table 1.** Scaling of jaw morphology over ontogeny in *Catoprion mento* and *Pygopristis denticulata.* For scaling scenarios, I = isometric growth, P = positive allometry and N = negative allometry.

independent variables	*r*^2^	isometric slope	intercept	slope	confidence intervals	*p*	scaling scenario	Δ slope	elevation? (*p =*)	shift?
Tooth Aspect Ratio_C_	0.243	**0**	0.622	0.125	0.070–0.224	5.16 × 10^−2^	**P**	0.16	<0.001	0.5
Tooth Aspect Ratio_P_	0.654	**0**	0.632	0.211	0.127–0.349	4.17 × 10^−3^	**P**			
Ant. MA_C_	0.386	**0**	0.700	−0.053	(−0.090)–(−0.031)	1.54 × 10^−2^	**N**	0.79	0.007	0.5
Ant. MA_P_	0.612	**0**	0.566	0.039	0.023–0.067	6.37 × 10^−3^	**P**			
Post. MA_C_	0.004	**0**	1.206	0.132	0.068–0.254	4.20 × 10^−1^	**P**	0.72	<0.001	<0.001
Post. MA_P_	0.206	**0**	1.020	0.111	0.054–0.231	1.10 × 10^−1^	**P**			
Occlusional Offset_C_	0.958	**1**	−0.737	1.057	0.916–1.220	1.60 × 10^−8^	**I**	0.68	0.01	0.15
Occlusional Offset_P_	0.995	**1**	−0.770	1.026	0.961–1.095	1.66 × 10^−9^	**I**			
Jaw Length_C_	0.986	**1**	0.397	0.983	0.905–1.067	5.94 × 10^−11^	**I**	0.001	<0.001	0.06
Jaw Length_P_	0.998	**1**	0.419	0.834	0.799–0.870	7.65 × 10^−11^	**N**			
Muscle CSA_C_	0.993	**2**	−1.998	1.946	1.770–2.139	6.50 × 10^−7^	**I**	0.28	<0.001	0.53
Muscle CSA_P_	0.983	**2**	−1.608	2.127	1.781–2.541	5.16 × 10^−5^	**I**			
Second Moment-0_C_	0.960	**4**	−10.735	4.964	4.311–5.751	1.33 × 10^−8^	**P**	0.13	0.65	0.20
Second Moment-0_P_	0.984	**4**	−9.779	4.365	3.901–4.885	7.44 × 10^−8^	**I**			
Second Moment-90_C_	0.974	**4**	−12.090	4.331	3.868–4.849	1.44 × 10^−9^	**I**	0.30	<0.001	0.84
Second Moment-90_P_	0.942	**4**	−9.186	3.861	3.121–4.776	6.79 × 10^−6^	**I**			

**Figure 4.**
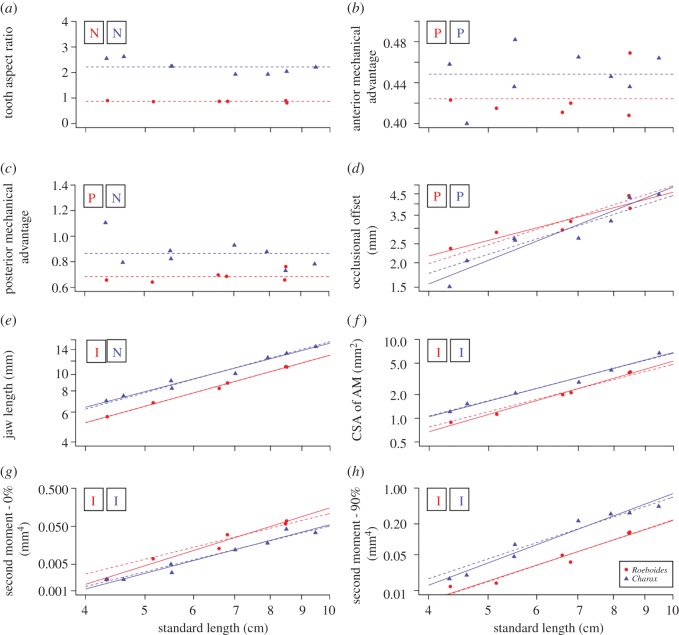
Reduced-major axis regressions of feeding morphology traits and standard length in *Roeboides affinis*, and *Charax* cf. *pauciradiatus* over ontogeny. (*a*) Tooth aspect ratio, (*b*) anterior mechanical advantage, (*c*) posterior mechanical advantage, (*d*) occlusional offset, (*e*) jaw length, (*f*) jaw adductor cross-sectional area, (*g*) second moment of area at the jaw joint (0%), (*h*) second moment of area at the jaw symphysis (90%). The dotted lines indicate theoretical isometric growth. Boxes represent the scaling pattern displayed by each species: N = negative allometry, P = positive allometry, I = isometric growth. Scale-feeders are outlined in red, their non-lepidophagous relatives outlined in blue.

For *Roeboides* and *Charax*, tooth aspect ratio scaled with negative allometry in both species (*Roeboides*: slope = −0.14, *Charax*: slope = −0.39; table 2, figure 4). Posterior mechanical advantage scaled with positive allometry in *Roeboides* and with negative allometry in *Charax* (*Roeboides*: slope = 0.23, *Charax*: slope = −0.43; table 2, figure 4), but the difference between these slopes was not found to be significantly different (*p* = 0.79). Jaw length grew isometrically in both species of characid fishes (*Roeboides*: slope = 0.99, *Charax*: slope = 0.94, figure 4). Scaling of jaw second moment of area scaled isometrically and did not differ at either the symphysis (*Roeboides*: slope = 5.05, *Charax*: slope = 4.24; table 2, figure 4) or the jaw joint (*Roeboides*: slope = 3.92, *Charax*: slope = 4.49). The adductor muscles of *Roeboides* and *Charax* also grew isometrically (*Roeboides*: slope = 2.25, *Charax*: slope = 2.04; table 2, figure 4).

**Table 2.** Scaling of jaw morphology over ontogeny in *Roeboides affinis* and *Charax* cf. *pauciradiatus.* For scaling scenarios, I = isometric growth, P = positive allometry and N = negative allometry.

independent variables	*r*^2^	isometric slope	intercept	slope	confidence intervals	*p*	scaling scenario	Δ slope	elevation? (*p =*)	shift?
Tooth Aspect Ratio_R_	0.164	**0**	1.098	−0.123	(−0.355)–(−0.043)	2.13 × 10^−1^	**N**	0.002	<0.001	<0.001
Tooth Aspect Ratio_C_	0.601	**0**	3.870	−0.891	(−1.615)–(−0.491)	1.19 × 10^−2^	**N**			
Ant. MA_R_	0.108	**0**	0.268	0.084	0.028–0.248	2.62 × 10^−1^	**P**	0.98	0.13	0.31
Ant. MA_C_	0.061	**0**	0.289	0.086	0.036–0.203	2.77 × 10^−1^	**P**			
Post. MA_R_	0.361	**0**	0.381	0.162	0.062–0.422	1.04 × 10^−1^	**P**	0.12	0.003	0.008
Post. MA_C_	0.303	**0**	1.604	−0.399	(−0.851)–(−0.187)	7.87 × 10^−2^	**N**			
Occlusional Offset_R_	0.837	**1**	−1.786	2.712	1.589–4.629	5.28 × 10^−3^	**P**	0.09	0.06	0.64
Occlusional Offset_C_	0.889	**1**	−3.625	3.546	2.556–4.919	2.25 × 10^−4^	**P**			
Jaw Length_R_	0.995	**1**	0.263	0.999	0.909–1.098	4.08 × 10^−6^	**I**	0.49	<0.001	0.62
Jaw Length_C_	0.977	**1**	0.543	0.949	0.815–1.105	2.04 × 10^−6^	**N**			
Muscle CSA_R_	0.989	**2**	−3.513	2.251	1.942–2.608	2.43 × 10^−5^	**I**	0.31	<0.001	0.82
Muscle CSA_C_	0.982	**2**	−2.791	2.045	1.697–2.466	6.35 × 10^−5^	**I**			
Second Moment-0_R_	0.964	**4**	−13.512	5.051	3.896–6.548	2.43 × 10^−4^	**I**	0.22	<0.001	0.61
Second Moment-0_C_	0.955	**4**	−12.679	4.245	3.437–5.244	1.48 × 10^−5^	**I**			
Second Moment-90_R_	0.948	**4**	−10.489	3.923	2.869–5.364	5.25 × 10^−4^	**I**	0.38	<0.001	0.55
Second Moment-90_C_	0.960	**4**	−10.592	4.492	3.683–5.478	1.02 × 10^−5^	**I**			

### 3.3. Similarities between lepidophagous taxa

Of the ontogenetic jaw mechanics traits shared between species pairs, both serrasalmids and characids, only isometric growth of the lower jaw and CSA of the adductor muscle were similar between scale-feeding fishes. Between *C. mento* and *R. affinis*, a similar trait found in common was tooth aspect ratio, which had values that were similar to each other (0.85 ± 0.02 s.e. and 0.86 ± 0.01 s.e., respectively), as well as these teeth being significantly stouter than their non-scale-eating counterparts. Additionally, the CSAs of the scale-feeding fishes were significantly smaller than their respective sister taxa.

Characid and serrasalmid species pairs showed elevational changes between their respective regression lines, i.e. where the ontogenetic trajectory of one species was higher than its sister taxon. *Pygopristis* and *Charax* had significantly more cuspidate teeth throughout their ontogeny relative to *Catoprion* and *Roeboides* (respectively), although the slopes of these relationships were indistinguishable between sister taxa (tables 1 and 2). A similar pattern was evident for muscle CSA, where non-lepidophagous taxa had conspicuously larger muscle masses consistently over ontogeny than their scale-feeding counterparts (tables 1 and 2). No other morphological similarities were apparent among the two lepidophagous taxa.

**Figure 5.**
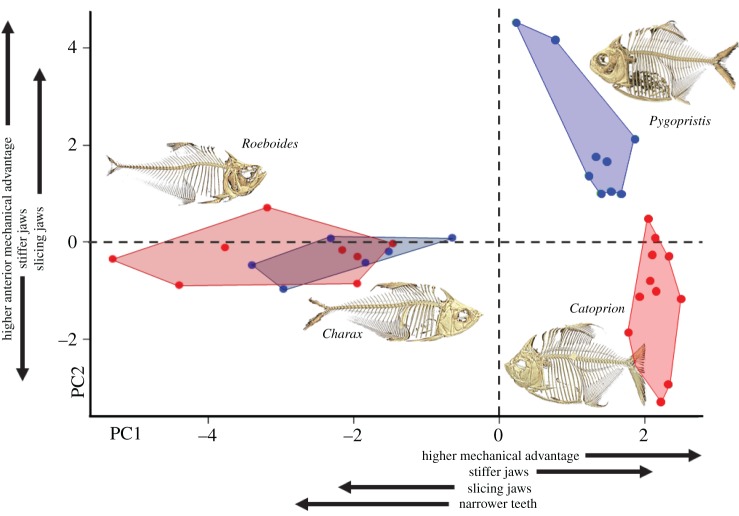
A principal component analysis of functional feeding traits from all characiform species in this study. Convex hulls are drawn around each species, points are individual specimens. Scale-feeder hulls are outlined in red, their non-lepidophagous relatives outlined in blue.

Scale-feeding taxa functionally resemble their sister taxon more closely than other scale-feeders. *Roeboides* and *Charax* largely overlap in their trait values, having similar mechanical advantages and jaw morphologies. The results of the PCA (electronic supplementary material, table S1) show that while individuals of *Catoprion* and *Pygopristis* generally do not overlap functionally, *Roeboides* and *Charax* have largely similar mechanical configurations (figure 5). The PCA loadings showed trends corroborated by ANOVA and regression results, e.g. *Charax* generally had narrower, more pointed teeth than *Roeboides*, which had broader, more robust teeth. Both characins had narrower teeth relative to the serrasalmid taxa. The average AMA of *Pygopristis* was greater than that of *Catoprion*; however, both serrasalmids had greater mechanical advantage than either characin taxa. *Pygopristis* had jaw occlusion suitable for slicing action, while *Catoprion* had jaws built for gripping or crushing. Both scale-feeders however had more robust jaws (more material distributed around a neutral axis in the *Y*-plane) than their non-lepidophage cousins.

## 4. Discussion

### 4.1. Is scale-feeding an ecomorphological monolith?

Mechanically and functionally, the lepidophagous fishes we examined have little in common. There is no archetypal morphology for scale-feeding as seen for some other dietary strategists like hard prey crushers (robust teeth and jaws, large jaw muscles), piscivores (large epaxial muscles, protrusible or tube-like mouths) and herbivores (multicuspid teeth, grinding dentition, long gut tract). Instead we find three different morphologies, one specialized for bodily ramming into prey to dislodge small scales (*Roeboides*), another which feed orally on small scales as a juvenile (*Pygopristis*) but move on to other prey as an adult, and a third which appears to feed on large scales consistently throughout its ontogeny (*Catoprion*) [23,36]. *Roeboides*' morphospace largely overlaps with its sister taxon *Charax*, with only a opisthognathous jaw covered by stout teeth distinguishing the two. This shared morphospace is not the same as that of the piranhas, which have completely non-overlapping morphospaces (figure 5). The many-to-one mapping scheme can be used to explain morphological diversity in ecological guilds [5], resulting from functional equivalency in phylogenetically-conserved systems, yet here we find different functional outcomes mismatched to an overly-broad ecological category.

**Figure 6.**
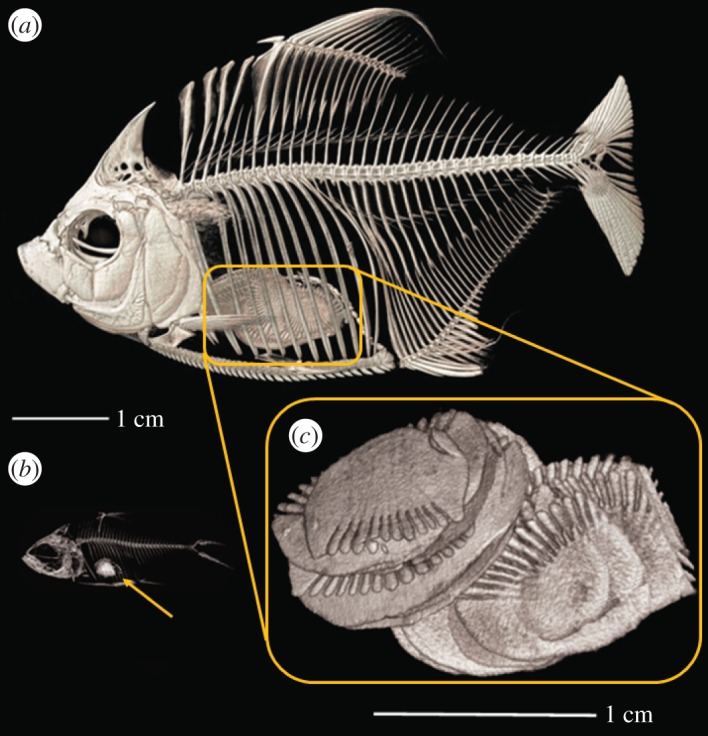
Scales in the gut of *Catoprion mento*. Adult (*a*) and juvenile (*b*) fishes have large scales (*c*) in their stomachs.

We believe these examples of lepidophagy are in fact very different niches from a functional and dietary point of view. The guts of *Catoprion* and *Roeboides* are packed with scales, but scales of very different size and number (figure 6). *Catoprion* eat large scales throughout their ontogeny [28,36]; in one specimen with 19 scales in its stomach, the average scale size was 80% the estimated maximum gape (assuming a gape angle of 120° [36]), and 14% larger than the length of the lower jaw. Larger scales are often found on larger fishes, which may be attractive to scale-feeders which retain a lepidophagous ecology as large adults [19]. Given the sort of niche partitioning diversity evident in ‘narrow-niche’ fishes like wood-eating catfishes [37], why should we not expect similar patterns to be replicated in the numerous scale-feeding fishes inhabiting similar habitats? Scale-feeding fishes typically inhabit an ecological niche-continuum spanning mucophages, and presumably pterygophages (perhaps even ectoparasite feeders [19,38]), facilitating specialization on any of these nuances of vertebrate ectoparasitism.

*Catoprion* is one of the few fish we know of that specialize in scales that are very large relative to their gape throughout ontogeny (figure 6, see *Terapon jarbua* [19]). As juveniles, wimple piranhas are one of many neotropical lepidophagous freshwater fishes, but as adults they alone among scale-feeders exploit the scales of comparably large prey [19,36,38]. This stands in contrast to lepidophagous characins, which consume small scales at small sizes and even when these characins reach larger sizes, continue to eat greater quantities of small scales rather than larger ones [19,39]. Adult *Catoprion* are also distinguished from most other scale-feeders (and other piranhas) in being a solitary predator, while even large *Roeboides* and *Terapon* are found interspersed among their prey or in shoals with conspecifics [38,40]. Juvenile lepidophagous characins occasionally mimic their prey, and are almost always found in schools. In contrast, adult *Catoprion* control specific territories [27,41], reflecting the need for access to large, mobile prey fishes that pass through these territories. The very traits that make piranhas excellent predators on fishes and fruits [42,43], wide gape, fast jaw closure, strong jaws, are exapted in *Catoprion* to lever scales from large fishes.

### 4.2. Development of scale-feeding morphologies

Ontogenetic slopes between lepidophage and non-lepidophage relatives show distinct differences in slope elevation while exhibiting few differences in actual slope. This pattern is consistent with the hypothesis that static allometries between species are difficult to evolve while allometric slope elevations are more readily evolvable [44–46]. In theory, natural selection can act more readily on slope elevations in static allometries because differences in elevation reflect differences in relative trait sizes within population means. *Roeboides* and *Charax* exhibit indistinguishable allometric slopes for tooth aspect ratio; however, the allometric elevation (intercept) shows lepidophagous *Roeboides* have markedly stouter teeth than piscivorous *Charax*. A similar pattern exists between *Catoprion* and *Pygopristis* with regards to tooth shape and posterior jaw leverage (mechanical advantage); *Catoprion* have markedly greater allometric elevation. These differences likely reflect the need of *Catoprion* for stout, robust teeth to remove scales and a greater posterior jaw leverage to close the jaw at extreme gape angles [36].

**Figure 7.**
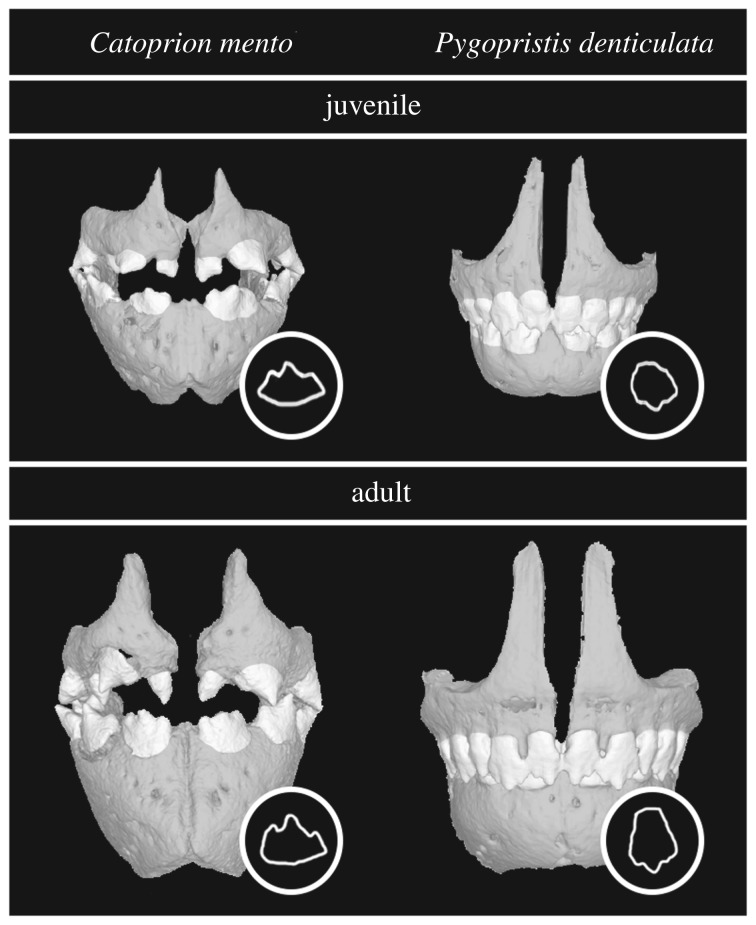
Comparison of tooth shape in *Catoprion mento* and *Pygopristis denticulata* as juveniles and as adults. Tooth outlines in lower right corner of each species' panel.

We find evidence for paedomorphosis (retention of juvenilized morphology) (*sensu* [47,48]) in the evolution and development of several associated feeding morphologies in *Catoprion* compared with its close generalist relative, *Pygopristis*. At juvenile stages both species exhibit similar tooth shapes. However, later in development *Pygopristis* grow longer teeth while *Catoprion* exhibit less pronounced dental growth (figure 7). The retention of juvenile tooth morphology in *Catoprion* is consistent with paedomorphosis. These juvenilized teeth may aid in lepidophagous habits as they exhibit a smaller aspect ratio, suggesting that the teeth are more resistant to breakage [43].

We might expect that these observed patterns of mosaic heterochrony allow scale-feeding fishes with diverse ancestral bauplans to converge on similar end phenotypes (i.e. odontocetes and crocodylians [49]). This is not the case, for across diverse lineages with diverse ancestral bauplans, lepidophagous fishes and other dietary specialists (e.g. compare piscivores [50,51]), fail to converge on similar functional, behavioural, or even ecological outcomes. Rather than invoke many-to-one mapping as a panacea for when morphology cannot predict ecology, we maintain that this mismatch lies instead with broad-brush attempts to reduce the functional diversity in natural history to ecological placeholders like guilds and trophic levels. Given that scale-feeding fishes exhibit variability in both the extent (ecology) and duration (ontogeny) of lepidophagy, these fishes offer a potent system for examining morphological and ecological specialization, and what relationships exist (or don't) between these paradigms.

### 4.3. A behavioural hypothesis for tooth form and function in lepidophagous fishes

Our data agree with prior studies, all demonstrating that shared morphological adaptations for scale-feeding (at least among Characiformes) involve specialized dentition [41,52]. *Catoprion* and *Roeboides* both attack their prey head on at roughly 90° angles, typically using ram attacks to dislodge scales [17,36]. African scale-feeding cichlids, *Perissodus straeleni* and *Perissodus microlepis*, have recurved laminar teeth that they use to laterally pry scales from prey [20,52]. These species maintain mouth-to-prey contact as they rotate along the long axis of their body to remove scales. Similarly, juvenile *Oligoplites* use hook-shaped teeth to attack prey from the rear, scraping scales parallel to the long axis of their prey's body [13,41]. The major difference between these two types of scale-feeding behaviours, perpendicular ram-feeding and orthogonal scraping, are distinguished by the tooth shape they require. Ram feeding requires stout teeth that withstand the impact, while scraping is facilitated by recurved teeth which pry scales from prey.

## Supplementary Material

Figure S1

## Supplementary Material

Table S1

## Supplementary Material

Table S2

## Data accessibility

Our code and data are deposited at Dryad (http://dx.doi.org/10.5061/dryad.q4157) [53]. CT scan data are available using the following MorphoSource identifiers: *Catoprion mento* (S6825, S8029, M16103, M16136, M17551), *Pygopristis denticulata* (M16105–16113), *Roeboides affinis* as *R. thurni* (M17594–17599, S9555) and *Charax* sp. as *C. pauciradiatus* (M20028).
