# Supplementary figures and images for: Specialized specialists and the narrow niche fallacy: a tale of scale-feeding fishes
Source: R Soc Open Sci. 2018 Jan 17;5(1):171581. doi: 10.1098/rsos.171581 (PMC5792939; doi:10.1098/rsos.171581)

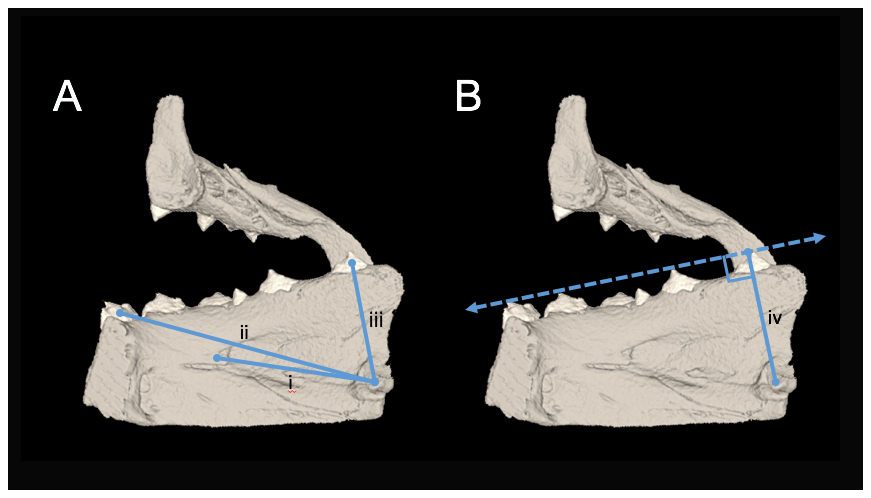

Supplement: Figure S1 [file rsos171581supp1.tif]
