# Supplementary material for: Specialized specialists and the narrow niche fallacy: a tale of scale-feeding fishes
Source: R Soc Open Sci. 2018 Jan 17;5(1):171581. doi: 10.1098/rsos.171581 (PMC5792939; doi:10.1098/rsos.171581)
Supplement: Table S1 [file rsos171581supp2.docx]

| Supplemental Table 1. PCA loadings of the functional feeding traits for the four characiform species | | | | | | | | | | | | | | |
| --- | --- | --- | --- | --- | --- | --- | --- | --- | --- | --- | --- | --- | --- | --- |
|  | Comp.1 | Comp.2 | Comp.3 | Comp.4 | Comp.5 | Comp.6 | Comp.7 | Comp.8 | Comp.9 | Comp.10 | Comp.11 | Comp.12 | Comp.13 | Comp.14 |
| Tooth Aspect Ratio | -0.283 |  |  | 0.562 | 0.384 | 0.211 | -0.164 |  | -0.176 | 0.530 | -0.159 | -0.195 |  |  |
| In-Lever |  | -0.157 | 0.606 |  |  |  |  |  |  |  |  |  | 0.615 | 0.450 |
| Ant. Out-Lever | -0.182 | -0.227 | 0.518 |  |  |  |  |  |  |  |  | 0.151 |  | -0.777 |
| Post. Out-Lever | -0.298 | -0.104 | 0.415 | -0.198 |  |  |  |  |  |  | 0.114 | -0.257 | -0.679 | 0.351 |
| Ant. MA | 0.365 | 0.180 | 0.160 | 0.138 | -0.303 |  | 0.208 |  | -0.237 | 0.189 |  | 0.434 | -0.568 | -0.210 |
| Post. MA | 0.374 |  | 0.159 | 0.229 |  |  | 0.446 | 0.175 | -0.132 | 0.273 | -0.113 | 0.536 | -0.363 | 0.138 |
| Occlusional Offset | 0.252 | -0.206 |  | -0.343 | -0.178 | 0.695 | -0.361 | 0.169 |  | 0.305 |  |  |  |  |
| Jaw Length | -0.124 | -0.467 |  | 0.418 |  | 0.301 | 0.102 | 0.354 | -0.137 | -0.518 | 0.251 |  |  |  |
| Jaw Max Height 0% | 0.407 |  | 0.147 |  |  |  | -0.154 |  | -0.270 | -0.345 | -0.693 | -0.313 |  |  |
| Jaw Max Width 0% | 0.300 | -0.203 |  | 0.176 |  | -0.502 | -0.664 | 0.214 |  |  | 0.236 | 0.132 |  |  |
| Jaw Max Height 50% | 0.341 | -0.122 | 0.149 | 0.330 |  | 0.190 |  | -0.596 | 0.566 | -0.144 |  |  |  |  |
| Jaw Max Width 50% |  | -0.246 | -0.152 |  | 0.244 | -0.816 | -0.120 | -0.178 | -0.197 | -0.125 | -0.259 |  |  |  |
| Jaw Max Height 90% |  | 0.537 | 0.217 | 0.118 |  | 0.235 | -0.273 | -0.236 | -0.423 | -0.293 | 0.258 | 0.364 |  |  |
| Jaw Max Width 90% | -0.109 | 0.497 | 0.188 | 0.239 | -0.176 | 0.100 |  | 0.547 | 0.530 |  | -0.136 |  |  |  |
|  |  |  |  |  |  |  |  |  |  |  |  |  |  |  |
| SS Loadings | 1.000 | 1.000 | 1.000 | 1.000 | 1.000 | 1.000 | 1.000 | 1.000 | 1.000 | 1.000 | 1.000 | 1.000 | 1.000 | 1.000 |
| Proportion Var | 0.710 | 0.710 | 0.710 | 0.710 | 0.710 | 0.710 | 0.710 | 0.710 | 0.710 | 0.710 | 0.710 | 0.710 | 0.710 | 0.710 |
| Cumulative Var | 0.071 | 0.143 | 0.214 | 0.286 | 0.357 | 0.429 | 0.500 | 0.571 | 0.643 | 0.714 | 0.786 | 0.857 | 0.929 | 1.000 |
