# Supplementary material for: Specialized specialists and the narrow niche fallacy: a tale of scale-feeding fishes
Source: R Soc Open Sci. 2018 Jan 17;5(1):171581. doi: 10.1098/rsos.171581 (PMC5792939; doi:10.1098/rsos.171581)
Supplement: Table S2 [file rsos171581supp3.docx]

| *Catoprion mento* | CAS | 20216 |
| --- | --- | --- |
| *Catoprion mento* | ROM | 85905 |
| *Catoprion mento* | ROM | 86225 |
| *Catoprion mento* | ROM | 85951 |
| *Catoprion mento* | ROM | 95239 |
| *Catoprion mento* | ROM | 86225 |
| *Catoprion mento* | UF | 189320 |
| *Catoprion mento* | UF | 37061 |
| *Catoprion mento* | UF | 189173 |
| *Catoprion mento* | UF | 77720 |
| *Catoprion mento* | UF | 81896 |
| *Catoprion mento* | UF | 84428 |
| *Catoprion mento* | UF | 84444 |
| *Catoprion mento* | UF | 189173 |
| *Catoprion mento* | UF | 33729 |
| *Charax sp (cf. pauciradiatus)* | AUM | 45439 |
| *Pygopristis denticulata* | AUM | 36164 |
| *Roeboides affinis* | AUM | 44838 |
